# Advances and challenges in modeling inherited peripheral neuropathies using iPSCs

**DOI:** 10.1038/s12276-024-01250-x

**Published:** 2024-06-03

**Authors:** Jonas Van Lent, Robert Prior, Gonzalo Pérez Siles, Anthony N. Cutrupi, Marina L. Kennerson, Tim Vangansewinkel, Esther Wolfs, Bipasha Mukherjee-Clavin, Zachary Nevin, Luke Judge, Bruce Conklin, Henna Tyynismaa, Alex J. Clark, David L. Bennett, Ludo Van Den Bosch, Mario Saporta, Vincent Timmerman

**Affiliations:** 1 Peripheral Neuropathy Research Group, Department of Biomedical Sciences, University of Antwerp, 2610 Antwerp, Belgium; 2 Laboratory of Neuromuscular Pathology, Institute Born Bunge, 2610 Antwerp, Belgium; 3 Institute of Oncology Research (IOR), BIOS+, 6500 Bellinzona, Switzerland; 4 Università della Svizzera Italiana, 6900 Lugano, Switzerland; 5 Universitätsklinikum Bonn (UKB), University of Bonn, Bonn, Germany; 6 Northcott Neuroscience Laboratory, ANZAC Research Institute Sydney Local Health District and Faculty of Medicine and Health, University of Sydney, Sydney, NSW Australia; 7 Molecular Medicine Laboratory, Concord Hospital, Sydney, NSW Australia; 8 UHasselt - Hasselt University, BIOMED, Laboratory for Functional Imaging and Research on Stem Cells (FIERCE Lab), Agoralaan, 3590 Diepenbeek, Belgium; 9 VIB-Center for Brain and Disease Research, Laboratory of Neurobiology, 3000 Leuven, Belgium; 10 Department of Neurology, Johns Hopkins University School of Medicine, Baltimore, MD USA; 11 Gladstone Institutes, San Francisco, CA USA; 12 Department of Pediatrics, University of California, San Francisco, San Francisco, CA USA; 13 Department of Ophthalmology, University of California, San Francisco, San Francisco, CA USA; 14 Department of Medicine, University of California, San Francisco, San Francisco, CA USA; 15 Stem Cells and Metabolism Research Program, Faculty of Medicine, University of Helsinki, 00290 Helsinki, Finland; 16 Blizard Institute, Barts and the London School of Medicine and Dentistry, Queen Mary University of London, London, UK; 17 Nuffield Department of Clinical Neuroscience, Oxford University, Oxford, UK; 18 Department of Neurosciences, Experimental Neurology, and Leuven Brain Institute, KU Leuven-University of Leuven, 3000 Leuven, Belgium; 19 Department of Neurology, Miller School of Medicine, University of Miami, Miami, FL USA

**Keywords:** Somatic system, Induced pluripotent stem cells

## Abstract

Inherited peripheral neuropathies (IPNs) are a group of diseases associated with mutations in various genes with fundamental roles in the development and function of peripheral nerves. Over the past 10 years, significant advances in identifying molecular disease mechanisms underlying axonal and myelin degeneration, acquired from cellular biology studies and transgenic fly and rodent models, have facilitated the development of promising treatment strategies. However, no clinical treatment has emerged to date. This lack of treatment highlights the urgent need for more biologically and clinically relevant models recapitulating IPNs. For both neurodevelopmental and neurodegenerative diseases, patient-specific induced pluripotent stem cells (iPSCs) are a particularly powerful platform for disease modeling and preclinical studies. In this review, we provide an update on different in vitro human cellular IPN models, including traditional two-dimensional monoculture iPSC derivatives, and recent advances in more complex human iPSC-based systems using microfluidic chips, organoids, and assembloids.

## Unveiling progress in modeling inherited peripheral neuropathies

Inherited peripheral neuropathies are diseases that cause damage to the motor and sensory nervous system. Despite progress in understanding these diseases, effective treatments are still hard to find. This study looks at using induced pluripotent stem cells (iPSCs - cells that can turn into any type of cell in the body) to mimic the disease and find possible drug targets. The scientists used iPSCs to create different nerve cells and Schwann cells (cells that support nerve function). They studied these cells to see how the disease affects them. The study found that models made from iPSCs can accurately copy key aspects of the disease, providing valuable insights that add to findings from animal models. This research could lead to new treatments for inherited peripheral neuropathies.

This summary was initially drafted using artificial intelligence, then revised and fact-checked by the author.

## Introduction

**Fig. 1:**
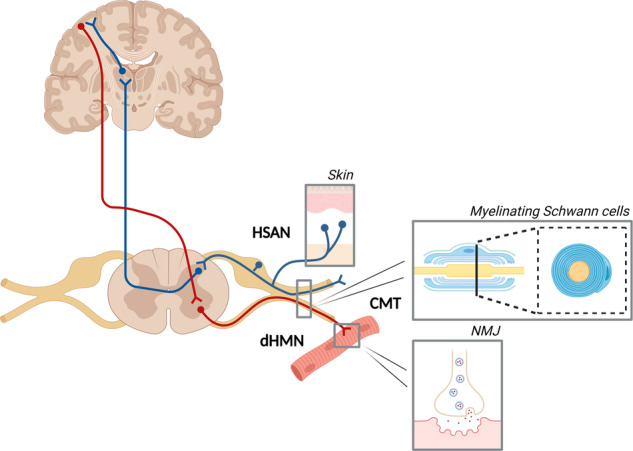
Schematic overview of the peripheral nerves and associated cell types involved in inherited peripheral neuropathies (IPNs). The scheme illustrates the different subtypes of IPN: Charcot-Marie-Tooth (CMT), which affects both motor and sensory neurons; hereditary sensory and autonomic neuropathy (HSAN), which affects only sensory and/or autonomic neurons; and distal hereditary motor neuropathy (dHMN), which affects only motor neurons. The boxes on the schematic represent known cell types that contribute to/act in IPN pathogenesis. NMJ neuromuscular junction. Created with BioRender.com.

Inherited peripheral neuropathies (IPNs) are a group of diseases characterized by the degeneration of motor and sensory nerves of the peripheral nervous system. Charcot-Marie-Tooth (CMT) neuropathy, also known as hereditary motor and sensory neuropathy (HMSN), is the most common IPN, affecting approximately 1 in 2500 people worldwide. Since it was first reported in 1886, progress in characterizing CMT has led to the identification of a clinically diverse and genetically heterogeneous group of disorders with more than 1000 mutations in more than 100 distinct CMT disease-associated genes^1,2^. Other IPN subtypes include hereditary motor neuropathy (HMN) and hereditary sensory and autonomic neuropathy (HSAN), in which motor and sensory neurons are affected, respectively (Fig. 1). Patients typically present with length-dependent degeneration primarily affecting the longest peripheral axons, resulting in progressive sensory loss, muscle weakness and atrophy of distal limb muscles^3^. CMT is broadly classified into demyelinating (CMT1) and axonal (CMT2) forms based on electrophysiological criteria and reflecting primary deficits occurring in myelinating Schwann cells or neuronal axons, respectively. Patients with intermediate CMT subtypes exhibit both demyelination and axonal pathology.

Over the past 10 years, significant advances in the biological understanding of the disease, using cell biology studies and transgenic rodent models, have led to the identification of several possible drug targets for IPNs. However, despite some encouraging preclinical results, the development of successful treatments for IPNs has remained challenging. Notably, there have been exceptional cases, such as the recent development of gene-silencing therapies for transthyretin amyloid neuropathy^4,5^. This breakthrough highlights the potential of targeted therapies for specific IPN subtypes. However, the challenges in translating preclinical success to clinical efficacy cannot be ignored. The low animal-to-human translational success rates, as well as the challenges in undertaking trials in a slowly progressive condition, such as CMT, in which outcome measures need to be optimized, must be considered. For example, high doses of ascorbic acid (vitamin C) resulted in reduced *PMP22* expression, accompanied by improvements in myelination, locomotion, and survival, in C22 mice overexpressing human *PMP22*^6^. For CMT1A patients, ascorbic acid treatment failed to result in a significant therapeutic benefit across multiple randomized clinical trials^7–10^. In contrast, oral L-serine supplementation was shown to reduce neurotoxic 1-deoxy-sphingolipid (1-deoxySL) production both in mice and in humans with *SPT*-associated HSN1 (HSAN-I)^11^. A pilot study including patients with the *SPTLC1*^*C133Y*^ mutation revealed that L-serine supplementation was associated with a reduction in 1-deoxySL levels^11^. Another 2-year trial demonstrated that L-serine was well tolerated and decreased 1-deoxySL levels^12^. Although the primary endpoint of this trial was not met, L-serine treatment resulted in notable improvements in patients, including patient-reported improvements in sensory symptoms and increased strength in both the upper and lower extremities during examination. However, it is important to note that L-serine treatment did not reduce the occurrence of skin ulcers, and there was a greater frequency of skin infections and osteomyelitis in the L-serine group than in the placebo group. Additional adverse events included localized abdominal pain and dyspepsia. These findings underscore the complexity of treating HSN1 (HSAN-I) mutation-related symptoms and highlight the need for alternative research approaches. To address the limitations faced in translating preclinical findings to successful treatments, it is essential to incorporate (novel) human cellular disease models that accurately replicate the key features of IPNs. By using induced pluripotent stem cells (iPSCs), which can be used to generate patient-specific cellular models, we can now study difficult-to-obtain cell types, such as differentiated Schwann cells and peripheral motor and sensory neurons, in a tailored and disease-relevant context. These advanced human cellular models possess the ability to (at least partially) bridge the existing gap and offer invaluable insights that not only complement but also augment the findings derived from animal models.

## Accessing IPN disease-impacted cells: the potential of iPSC technology

### Peripheral motor and sensory neurons

Since the discovery of iPSCs, these cells have become the workhorse for developing tissue-relevant disease preclinical models. The groundbreaking work of reprogramming mature cells into a pluripotent state^13^ has led to invaluable contributions to the field of regenerative medicine. In 2009, familial dysautonomia (FD), also known as Riley-Day Syndrome or HSAN-III, became the first IPN modeled through human PSC-derived lineages in vitro^14^. The IPN field has also leveraged this essential technology, with significant efforts focused on in vitro modeling using motor neurons. In 2015, Saporta et al. published their work on the first iPSC model, describing the cellular phenotypes of two axonal CMT subtypes^15^. In the following years, numerous inherited neuropathy disease-causing mutations/subtypes as well as acquired neuropathies have been modeled using iPSCs^16^.

A wide range of protocols is currently available for deriving and studying motor neurons. Table 1 provides an overview of the differentiation protocols used to generate iPSC-derived motor neurons harboring IPN mutations. Most protocols consist of small molecule compounds that rely on dual SMAD inhibition for neural induction, including LDN193189 (a BMP signaling inhibitor) and SB431542 (an activin-nodal signaling inhibitor). Studies have also used alternative BMP inhibitors, such as dorsomorphin^17,18^. For some protocols, dual SMAD inhibition is combined with CHIR99021 (WNT activation) to promote posterior identities and neuroepithelial proliferation^19–21^ or with FGF2 to enhance the neural differentiation of pluripotent stem cells^22–24^. After neural induction, retinoic acid (RA) and recombinant SHH (Sonic Hedgehog) or its agonist, SAG (Smoothened Agonist), are commonly used to promote caudal and ventral patterning toward a motor neuron fate. In addition, some protocols^21^ include supplementation with a potent γ-secretase inhibitor DAPT, which is known to promote neurogenesis and rapidly drive all progenitors into postmitotic neurons within a few days^19^. However, it is essential to note that the classification of motor neurons as peripheral nervous system or central nervous system (CNS) neurons may vary depending on the perspective. Motor neurons, derived from the neurectoderm, have cell bodies located in the spinal cord (Fig. 1), which aligns with a CNS classification. Nevertheless, in the context of IPNs, we emphasize their significant role in axonal degeneration within the peripheral nervous system.

**Table 1:** Overview of all iPSC-derived motor neuron protocols used in IPN.

Reference	SMAD inhibition	Patterning	Inclusion of DAPT
Hu et al., ^158^	-	RA, SHH	-
Reinhardt et al., ^159^	SB, DM, CHIR	RA, PMA	-
Saporta et al. ^15^	SB, DM	RA, SAG	-
Maury et al. ^19^ & adapted in Guo et al. ^20^	LDN, SB, CHIR	RA, SAG	Yes
Amoroso et al., ^160^	LDN, SB	RA, PMA	-
Faye et al. ^22^	SD, DM, Noggin, FGF2	RA, SHH	-
Fernandopulle et al., ^161^	Induced expression of the transcription factors NGN2, ISL1, and LHX3 (‘NIL’)		

In recent years, an increased generation of inducible iPSC lines has been developed. The iPSC lines express the human transcription factors *NGN2*, *ISL1*, and *LHX3* (‘hNIL’) (with the hNIL construct inserted into a safe harbor locus) under the control of doxycycline^25^. After 3 days of differentiation, doxycycline was added, resulting in the rapid generation of motor neurons with high purity. In contrast to small molecule-based protocols, this protocol involves the continuous high expression of transcription factors instead of stepwise motor neuron development. Recently, the overexpression of the transcription factor Neurogenin2 (*NGN2*) was combined with small molecule patterning to differentiate iPSCs into lower induced motor neurons, demonstrating high reproducibility in multiple cell lines and successful production of disease-relevant motor neuron populations^26^. However, it is essential to note that the overexpression of transcription factors, as employed in some protocols, may mask developmental defects and/or pathogenic mechanisms.

However, despite these advancements in motor neuron differentiation protocols, challenges related to variability and heterogeneity in motor neuron identity and purity persist. The use of different differentiation protocols can significantly influence the efficiency and maturity of the resulting motor neurons. In the context of IPNs, this issue becomes particularly complex, as IPN motor neuron studies stem from seven distinct ‘reference’ protocols (Table 1). This diversity poses a significant hurdle when comparing and interpreting research findings. Heterogeneity is another critical aspect to consider, as differentiation protocols could give rise to mixed populations of cells, which can hinder motor neuron purity and functionality. To address this, additional steps have been undertaken within the different protocols reported to obtain higher purity cultures. Saporta et al. reported a sorting step (bead-based enrichment for L1CAM) to eliminate non-neuronal cells^15^. Other protocols have included the use of SN38-P to remove proliferative stem cells and neuroprogenitors^17,18,27^ or Ara-C to prevent non-neuronal dividing cells^21,28^. Notably, the inclusion of an Hb9:GFP reporter in protocols offers a targeted approach to enhance the identification and increase the purity of motor neurons^26^.

The generation of sensory neurons has undergone similar protocol developments. In IPN research, all iPSC-SN studies^21,28,29^ have used (with or without modifications) the small compound protocol developed by Chambers et al. ^30^, which yields nociceptive sensory neurons^30^. However, it is crucial to recognize that within general sensory neuron populations, a diverse array of subtypes exist, including proprioceptive, mechanoreceptive, and nociceptive neurons, each contributing to overall sensory function. Human iPSCs can now be differentiated into mechanosensing sensory neurons^31^ or nociceptors, either by employing small molecule pathway inhibitors^30,32^ or through transcriptional programming via controlled coexpression of *NGN2* and *BRN3A*^33^. However, generating all three sensory neuron subtypes at once has proven challenging^30,31,34^. Nevertheless, Saito-Diaz et al. ^35^ successfully produced all three subtypes^35^ in proportions similar to those observed in DRGs^36^. Moreover, by using an immunopanning approach, a gentle antibody-based purification technique, TRKA^+^ nociceptors, TRKB^+^ mechanoreceptors, and TRKC^+^ proprioceptors were isolated from bulk day-25 sensory neuron cultures, allowing specific investigation of different sensory neuron subtypes derived from one general sensory neuron culture. Still, the generation of distinct subpopulations of nociceptors from iPSCs remains elusive. Nociceptors produced using the Chambers protocol do not fully mature into adult nociceptors, as evidenced by the expression pattern of voltage-gated sodium channels, specifically NaV1.8 and NaV1.9^37,38^. There is substantial functional diversity and polymodality within the nociceptive neuron subpopulation. Recent advances in differentiation protocols for nociceptors have aimed to better model native nociceptors^39^; however, this challenge could be addressed through the use of more complex cellular models.

In HSAN, autonomic neurons and sensory neurons are involved in disease pathogenesis. These issues have been understudied due to the lack of a proper model system. Recently, protocols for generating iPSC-derived sympathetic neurons have been reported using gene-specific reporter lines^40^ or via the generation of neural crest cells, which are then further differentiated into sympathetic neurons^41–43^. Parasympathetic neurons also play important roles in diseases such as autonomic neuropathy and were recently differentiated by first passing through iPSC-Schwann cell progenitors (SCPs)^44^. Among IPNs, FD currently represents the sole condition in which peripheral autonomic neurons have been effectively modeled using human iPSCs^43,44^. The development of protocols to cogenerate motor, sensory, and autonomic iPSC-derived neuronal cell types will be the cornerstone in addressing the role of disease-causing IPN genes underlying motor, sensory, and/or autonomic neuron dysfunction. However, the comparison of such protocols might present challenges due to inherent variations in factors such as timing and procedural steps.

### Schwann cells and their precursors

Schwann cells play a key role in maintaining metabolic support to peripheral neurons by producing myelin, a lipid-rich structure that ensheathes associated axons. The vast majority of IPN patients have mutations in myelin-associated genes, which can impair the development, maintenance, and functional capacity of Schwann cell myelination. However, the generation of mature Schwann cells and functional in vitro human myelin has far more eluded researchers than the generation of other cells owing to heterogeneously differentiated cell populations, protocol reproducibility issues^45^, the influence of culture conditions^46,47^ and the low efficiency of human Schwann cell in vitro myelination, even with primary human Schwann cells. Therefore, research has focused mainly on the generation and utilization of Schwann cell precursors (SCPs) as a research tool and a source of iPSC-Schwann cells, which have been recently successfully demonstrated^48–51^. While SCPs hold promise, recognizing their limitations is essential. Producing fully mature Schwann cells from stem cells remains an important objective, providing deeper insights into myelination and disease.

### In vitro limitations: untangling the intertwined developmental cell fates of Schwann cell precursors

The Schwann cell lineage is closely interconnected to other neural crest-derived cells, such as melanocytes^52^. When neural crest cells first contact and associate with motor neurons and dorsal root ganglion (DRG) axons, they differentiate into SCPs through a process regulated by the transcription factor SOX10^53,54^. Developmental studies have shown that SCPs are multipotent stem cells capable of generating several peripheral tissue cell types, such as parasympathetic neurons, muscle cells, and melanocytes, in addition to Schwann cells^52,55–57^ (reviewed in Jessen & Mirsky^58^). The SCPs use peripheral nerve axons as guidance tracks to arrive at the most distal regions of the body and differentiate into mature myelinating Schwann cells (reviewed in Jessen & Mirsky^59^). Here, they remain for the lifetime of the individual, unless injury occurs (reviewed in Stassart & Woodhoo^60^). However, once SCPs dissociate from the peripheral nerve axons, they can differentiate into melanocytes via the upregulation of the cell fate determinant transcription factor microphthalmia (MITF)^52^. Separating the early melanocyte lineage, i.e., melanoblasts, from the Schwann cell lineage via in vitro differentiation protocols is difficult because their cell fate is regulated by the expression levels of common transcription factors, such as *FOXD3*^61^ (reviewed in Van Raamsdonk & Deo^62^), and pathways, such as the WNT/β-catenin pathway^63^. Similarly, early melanocyte lineage cells can express several Schwann cell proteins and RNA transcripts from genes such as *ErbB3*, *P75*, *SOX10*^64^, *PLP1,* and *MBP* (Golli-MBP RNA transcripts)^65,66^ and have bi- and tripolar morphologies. Therefore, untangling these two cell fates via in vitro differentiation has proven difficult. Recently, published iPSC-Schwann cell protocols demonstrate melanocyte lineage gene expression in their RNA sequencing profiles^47,49^.

## Insights into IPN pathomechanisms using iPSC models

**Fig. 2:**
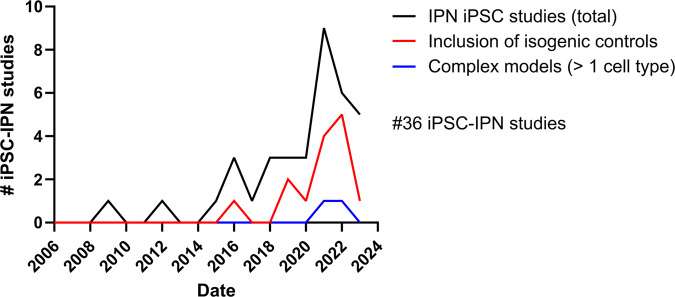
Graph showing all the IPN patient-specific iPSC papers published over time. The figure shows the IPN studies published over time (black), studies with isogenic controls (red), and studies examining multiple cell types (blue). The total number of studies, including preprints, is 36, all of which are listed in Table 2.

The use of iPSCs has revolutionized the study of IPNs by providing valuable insights into the underlying disease mechanisms associated with specific gene mutations. One of the key advantages of iPSC-derived models is the preservation of the human genetic background, allowing researchers to study relevant tissues that are often inaccessible for research purposes. Table 2 summarizes the current published models, with phenotypes and, if applicable, applied treatments, for human in vitro IPN research. Additionally, Fig. 2 visually represents the trends in published IPN patient-specific iPSC papers over time.

**Table 2:** Overview of current published IPN stem cell research and phenotypes.

IPN subtype	Affected genes & mutation	Human iPSC-derived cell model	Inclusion of isogenic line(s)	Phenotypical features? Treatment tested?	References
*CMT*					
CMT1A	*PMP22_dup*	iPSC-derived Schwann cell precursors	NA	Developmental disabilities of Schwann cells.	Shi et al., ^162^
CMT1A	*PMP22_dup*	iPSC-derived Schwann cell precursors	Yes	Upregulation of *CXCL1* and *MCP-1* proteins, as well an associated infiltration of immune cells into nerve tissues.	Mukherjee-Clavin et al. ^50^
CMT1A	*PMP22_dup*	iPSC-derived organoids	Yes	Early ultrastructural myelin alterations; increased myelin periodic line distance and hypermyelination of small axons. *Treatment?* shRNA & combinatorial treatment with baclofen, naltrexone hydrochloride, and D-sorbitol	Van Lent et al. ^106^
CMT1A	*PMP22_dup*	iPSC-Schwann cell precursors	Yes	Lipid metabolic abnormalities primarily associated with cholesterol and sphingolipids.	Prior et al. ^51^
CMT1A, CMT1B, CMT1D	*PMP22_dup, MPZ*^*R98C*^*, EGR2*^*R353G*^	iPSC-derived neural crest cells	NA	Glutathione-mediated detoxification gene expression pathway as common pathway behind demyelinating neuropathies.	Kitani-Morii et al., ^163^
CMT1F	*NEFL*^A367*^	iPSC-derived motor neurons	NA	Complete absence of NEFL protein.	Sainio et al., ^164^
CMT1F	*NEFL*^A367*^, NEFL^*−/−*^	iPSC-derived motor neurons	Yes	Absence of NEFL protein. Also, reduced axonal caliber, decreased amplitude of miniature excitatory postsynaptic currents, reduced NEFH levels, and no compensatory increases in other filament subunits. The movement of mitochondria and to a lesser extent lysosomes was increased. *Tested:* Nonsense suppressor drugs did not rescue NEFL loss.	Sainio et al., ^165^
CMT2A, CMT2E	*MFN2*^*R364W*^*, NEFL*^*N98S*^	iPSC-derived motor neurons	NA	Abnormal cytoskeletal and mitochondrial dynamics. Hyperexcitable and altered sodium and calcium channel kinetics.	Saporta et al. ^15^
CMT2A	*MFN2*^*A383V*^	iPSC-derived motor neurons	NA	Global reduction in mitochondrial content and altered mitochondrial positioning. Abnormal apoptosis resistance and increased autophagy. *Treatment?* RNAi/gene therapy combined approach	Rizzo et al., ^166^; Rizzo et al. ^151^
CMT2A, CMT2E	*MFN2*^*R94Q*^*, NEFL*^*P8R*^	iPSC-derived motor neurons	NA	Reduced *PFN2* expression	Juneja et al. ^112^
CMT2A, CMT2E, CMT2F, CMT2L	*MFN2*^*R94Q*^*, NEFL*^*P8R*^*, HSPB1*^*G84R*^*, HSPB1*^*P182L*^*, HSPB8*^*K141N*^	iPSC-derived motor and sensory neurons	Yes	Gene-specific phenotypes and common hallmarks of axonal degeneration such as impairments in axonal transport and mitochondrial function. *Treatment?* GNE-8505 (DLK-inhibitor)	Van Lent et al. ^21^
CMT2A	*MFN2*^*R94Q*^	Human ESCs-derived motor neurons	Yes	Impaired mitochondrial trafficking and reduced numbers of mitochondria in distal parts of axons. *Treatment?* ACY-738 (HDAC6 inhibitor)	Butler et al. ^86^
CMT2B	*RAB7A*^*V162M*^	iPSC-derived sensory neurons	NA	Increased lysosomal protein expression and lysosomal activity.	Romano et al. ^29^
CMT2D	*GARS1*^*P724H*^	iPSC-derived motor neurons	Yes	Deficiencies in spontaneous action potential firing and burst fire behavior. Decreased acetylated α-tubulin levels and mitochondrial movement within axons. *Treatment?* Tubastatin A and CKD504 (HDAC6 inhibitors)	Smith et al., 2021^167^
CMT2E	*NEFL*^*N98S*^	iPSC-derived motor neuron spheroids	NA	NEFL deposits and increased neurofilament levels in the culture supernatant. *Treatment?* PLK1 (PLKi) and CK2 inhibitor (kinase inhibitors)	Maciel et al. ^73^
CMT2E	*NEFL*^*N98S*^	iPSC-derived (inducible) motor neurons	Yes	Pathologic accumulation of NEFL protein in the cell body.	Feliciano et al. ^25^
CMT2F	*HSPB1*^*S135F*^*, HSPB1*^*P182L*^	iPSC-derived motor neurons	NA	Decreased acetylation of α-tubulin and axonal movement defects of mitochondria. *Treatment?* CHEMICAL X4 and CHEMICAL X9 (HDAC6 inhibitors)	Kim et al. ^146^
CMT2F	*HSPB1*^*P182L*^	iPSC-derived motor neurons	NA	Decreased autophagic flux.	Haidar et al. ^71^
CMT2F	*HSPB1*^*P182L*^	iPSC-derived motor neurons	NA	Large, cytoplasmic aggregates.	Alderson et al. ^72^
CMT2H	*GDAP1*^S194*^	iPSC-derived motor neurons	NA	Lipid dysfunction, oxidative stress, and mitochondrial cristae defects.	Miressi et al. ^24^
CMT2-AR	*SORD*^*−/−*^	iPSC-derived motor neurons	NA	Increased sorbitol levels. *Treatment?* AT-007 (govorestat) (Aldose reductase inhibitor)	Zhu et al. ^69^
CMT2	*MT-ATP6* ^*m.9154C>T*^	iPSC-derived motor neurons	Yes	Impaired assembly of ATP synthase, disrupted mitochondrial cristae morphology, defective differentiation with high heteroplasmy, metabolic shift with lower heteroplasmy.	Kenvin et al. ^74^
CMT4A	*GDAP1*^*(L239F/R273G)*^ *GDAP1*^*(c.579* *+* *1G>A)*^	iPSC-derived motor neurons	NA	Increased glutaminolysis, reduced lipid droplets, and reduced mitochondrial Ca^2+^ levels.	Wolf et al. ^168^
CMTX6	*PDK3*^*R158H*^	iPSC-derived motor neurons	Yes	Increased phosphorylation of the PDC, energy metabolism defects, and mitochondrial abnormalities. *Treatment?* DCA (pan PDK inhibitor)	Perez-Siles et al. ^17^
*dHMN*					
dHMNX	*ATP7A*^*T994I*^	iPSC-derived motor neurons	NA	Reduced ATP7A protein levels in the soma and failure to upregulate expression of ATP7A under copper-loading conditions.	Perez-Siles et al. ^27^
dHMN	*DHMN1*^*UBE3C-IF*^	iPSC-derived motor neurons	NA	Significant reduction of wild-type full-length UBE3C protein levels.	Cutrupi et al. ^18^
*HSAN*					
HSAN-I (HSN1)	*SPTLC1*^*C133W*^	iPSCs, iPSC-derived sensory neurons, and myelinating cocultures (iPSC-SN and rat Schwann cells)	NA	Production of neurotoxic deoxysphingolipid bases (DSBs), reduced complex gangliosides, and impaired neurotrophin signaling, resulting in reduced neurite outgrowth. In HSN1 myelinating cocultures, major disruption of nodal complex proteins causing myelin breakdown. *Treatment?* L-serine supplementation	Clark et al. ^28^
HSAN-III (Familial dysautonomia)	Homozygous *IKBKAP*^*IVS20+6* *T>C*^	iPSC-derived neural crest precursors	NA	Low levels of normal IKBKAP transcript. Decreased rate of neurogenesis, and reduced migration. *Treatment?* Early stage long-term kinetin	Lee et al. ^14^
HSAN-III	Homozygous *IKBKAP*^*IVS20+6T>C*^	iPSC-derived neural crest precursors	NA	Low levels of normal IKBKAP transcript and reduced migration. *Treatment?* SKF-86466	Lee et al., ^169^
HSAN-III	Homozygous *IKBKAP*^*IVS20+6T>C*^ *Severe HSAN-III patients had additional +/-mutations in *LAMB4*, *FAT2* or *KIAA1211*	iPSC-derived neural crest, and sensory and autonomic-like neurons	Yes	IKBKAP missplicing, deficit in *ASCL1* expression levels, and migration defects. Severe FD patient cells display impaired specification of NC derivatives, affecting autonomic and sensory neurons. Both severe and mild FD cells exhibit defects in peripheral neuron survival. *Treatment?* Kinetin and SKF-86466	Zeltner et al., ^170^
HSAN-III	Homozygous *IKBKAP*^*IVS20+6T>C*^	iPSC-derived neural crest and iPSC-derived sensory neurons	Yes	Affected dense ridges in NCC generation, decrease in SOX10, affected sensory neuron generation from iPSCs.	Saito-Diaz et al. ^35^
HSAN-III	Homozygous *IKBKAP*^*IVS20+6T>C*^	iPSC-derived sensory neurons	NA	Absence of serine/arginine-rich splicing factor 6 (SRSF6) binding to an intronic splicing enhancer in intron 20 *Treatment?* Small molecule splice modulator, RECTAS, interacts with CDC-like kinases (CLKs) and enhances SRSF6 cellular activity	Ajiro et al., ^171^
HSAN-III	Homozygous *IKBKAP*^*IVS20+6T>C*^	iPSC-derived neural crest, and sympathetic autonomic neurons	Yes	Spontaneous, intrinsic hyperactivity, defective norepinephrine autoregulatory pathway, lower NET expression. *Treatment?* Dexmedetomidine, carbidopa, clozapine, flupiritine, …	Wu et al. ^43^
HSAN-III	Homozygous *IKBKAP*^*IVS20+6T>C*^	iPSC-sympathetic and parasympathetic autonomic neurons	NA	FD-parasymANS are spontaneously hyperactive and the normal crosstalk with symANS is disconnected.	Wu et al. ^44^
Congenital insensitivity to pain (CIP)	Compound heterozygote *SCN9A*^*R830X & FS1773*^, *SCN9A*^*R896W & c.377+5C>T*^ *SCN9A*^*−/−*^	iPSCs, iPSC-derived sensory neurons	Yes	Nociceptors are hypoexcitable to both threshold and suprathreshold electrical stimuli *Treatment?* PF-05089771, BIIB074 (lacks specificity for Nav1.7, the protein product of SCN9A)	McDermott et al. ^70^

*Table also contains preprints.

**Fig. 3:**
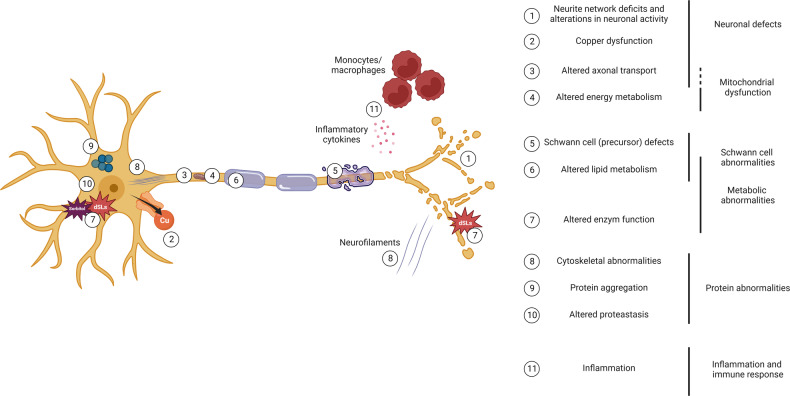
Illustration of a peripheral nerve and reported phenotypes described in inherited peripheral neuropathies (IPNs) using iPSCs. The schematic shows different phenotype terms, numbered and located within the neuron based on the reported IPN iPSC studies. These terms can then be broadly divided into the following categories: neuronal defects, mitochondrial dysfunction, Schwann cell, metabolic and protein abnormalities, inflammation, and immune response. Created with BioRender.com.

These IPN phenotypes can be broadly divided into the following categories: neuronal defects, mitochondrial dysfunction, Schwann cell abnormalities, metabolic irregularities, protein homeostasis abnormalities, and inflammatory immune responses (Fig. 3). Several of the modeled genes directly impact the categories defined by the cellular phenotypes.

### Modeling iPSC-motor, sensory, and autonomic neurons

iPSC-derived motor neurons carrying the *GARS*^P724H^ mutation causing CMT2D exhibit synaptic dysfunction, characterized by alterations in spontaneous action potential firing and burst behavior^67^. Another example involves mutations in the copper-transporting P-type ATPase (*ATP7A*) gene, which causes X-linked distal hereditary motor neuropathy (dHMNX)^68^. Patient-derived motor neurons harboring the p.T994I mutation exhibit reduced ATP7A expression and an impaired response to Cu-dependent ATP7A accumulation in dHMNX motor neurons^27^. Metabolic dysfunction has been exemplified by altered sorbitol levels in *SORD*^−/−^ motor neurons^69^ or the production of neurotoxic deoxysphingolipids in HSN1 sensory neurons associated with mutations in serine-palmitoyltransferase (SPT) subunits (SPTLC). These primary metabolic defects can have complex effects. For instance, toxic gain-of-function mutations in SPT subunits not only result in increased deoxysphingolipids but also impair the production of complex gangliosides in HSN1, with an impact on membrane composition and cell signaling^28^. iPSC nociceptors derived from congenital insensitivity to pain (CIP) patients exhibit impaired electrogenesis with reduced action potentials in response to depolarizing stimuli, demonstrating that NaV1.7 is a key regulator of excitability^70^. In addition to FD models showing neural crest and sensory neuron generation deficits, MEA analysis of iPSC-parasympathetic autonomic neurons demonstrated hyperactivity compared to the controls; however, the level of hyperactivity was not as high as that of iPSC-sympathetic autonomic neuron hyperactivity in FD^44^. These results mimic parasympathetic phenotypes in FD patients, which are subtler than sympathetic phenotypes. The modeling of genetic mutations involved in maintaining protein homeostasis, such as the *HSPB1*^P182L^ mutation, has been found to affect autophagy^71^ and cause large, cytoplasmic aggregates^21,72^. The *NEFL*^N98S^ mutation not only results in the accumulation of NFL within motor neurons but also the release of NEFL^73^. Finally, mitochondrial dysfunction has emerged as a prominent feature of various IPNs. For instance, the missense mutation p.R158H in the pyruvate dehydrogenase kinase 3 gene (*PDK3*) causes CMTX6. This mutation leads to the hyperphosphorylation of specific Ser residues of the pyruvate dehydrogenase complex (PDC), leading to reduced activity of PDC in patient-derived motor neurons and impacting energy metabolism^17^. Another interesting example arises from mutations in one of the mitochondrial DNA genes, *MT-ATP6*, encoding a subunit of ATP synthase, which can also cause CMT2. The modeling of a heteroplasmic MT-ATP6 nonsense mutation in patient iPSC-derived motor neurons showed that the assembly of ATP synthase was impaired and that mitochondrial morphology was subsequently distorted because the dimerization of ATP synthase is required for crista formation^74^. This links *MT-ATP6* to many other CMT2-related genes by altering mitochondrial dynamics in motor neurons. Notably, IPN phenotypes may also arise from combined defects in multiple categories, highlighting the interrelationships and interactions among various underlying cellular and molecular processes.

### Modeling the Schwann cell lineage

Focusing on the Schwann cell lineage, a recent study utilized iPSC-SCPs to model the lipid metabolic phenotype in CMT1A patient iPSCs^51^. The authors demonstrated that lipid metabolic defects occur early in the Schwann cell lineage and are characterized by mishandling of cholesterol and lipids in lipid droplets and late endosomes/lysosomes, resulting in reduced cholesterol incorporation into the plasma membrane of patient cells^51^. This homogenous iPSC-SCP model serves as a valuable model for studying early developmental abnormalities, such as mutations in *PMP22* and *MPZ*, in CMT1 patients^51^. Another recent study demonstrated immune dysregulation in both iPSC-SCPs and iPSC-derived Schwann cells (CXCL1 and MCP-1 overexpression) from CMT1A patient lines across different stem cell lines and differentiation protocols^50^.

Expanding the scope of models to related conditions, iPSC-based research has been instrumental in creating models of complex disorders such as neurofibromas and schwannomas, which share intricate links to specific genetic mutations. For instance, iPSCs have been used to mimic plexiform neurofibromas (PNFs) resulting from NF1 inactivation. In these studies, NF1^−/−^ iPSCs differentiated into Schwann cells exhibited a sustained high proliferation rate, compromised myelination capacity, and a tendency to form 3D spheres expressing markers akin to their PNF-derived primary Schwann cell counterparts^75^. Understanding these in vitro developments holds promise for furthering the understanding of the multifaceted composition of neurofibromas, comprising various cell types such as Schwann cells, endoneurial fibroblasts, perineurial cells, and infiltrating immune cells. This broader exploration not only enhances our grasp of the pathogenesis of peripheral nerve tumors but also provides insights into the fundamental biology of distinct cell types within peripheral nerves and the signaling among them.

### Modeling structural variations

While many iPSC-derived motor neuron models harbor patient point mutations in genes, structural variation (SV) mutations are also crucial players in IPN pathogenesis. Structural variation is a broad term encompassing genomic rearrangements that disrupt the chromosomal organization and genome architecture^76^ and can range in size from 50 base pairs to millions of base pairs^77^. Two large complex SV events have been reported to cause DHMN1^78^ (OMIM: %182960) and CMTX3^79^ (OMIM: %302802). Modeling the pathogenic effects of SV mutations on peripheral nerves is challenging. However, leveraging iPSC technologies to model SV mutations in patient-derived motor neurons has been a powerful strategy for studying disease-relevant tissue while preserving the genetic background of the SV. This strength is exemplified by recent work from Cutrupi et al. ^18^, in which an iPSC-derived spinal motor neuron model was generated from DHMN1 patients harboring a complex 1.35 Mb intrachromosomal insertion. Using a combination of targeted and global transcriptomics in conjunction with cutting-edge chromatin conformation capture studies, the authors excluded neuronal-specific gene dysregulation as a potential disease mechanism and discovered a novel gene-intergenic fusion transcript involving the ubiquitin E3 ligase gene *UBE3C*. The novel transcript (*UBE3C-IF*) does not undergo nonsense-mediated decay, which results in a significant reduction in wild-type full-length UBE3C (UBE3C-WT) protein levels in DHMN1 iPSC-derived spinal motor neurons^18^. Future investigations using this model can now focus on exploring strategies to eliminate *UBE3C-IF* with the goal of restoring normal UBE3C function and abrogating disease progression.

### Common phenotypes?

Remarkably, studies modeling these and other IPN mutations using iPSC-derived motor and sensory neurons have also described mitochondrial dysfunction (Table 2). A recent study demonstrated progressive axonal transport and mitochondrial deficits in five CMT2 patient iPSC-derived motor neurons. In contrast, dHMN subtypes did not exhibit mitochondrial defects when the same iPSCs were differentiated into sensory neurons^21^. Mitochondrial deficits and altered transport have also been observed in several CMT mouse models^80^, as well as in other cellular IPN models, including primary motor neuron axons expressing wild-type and mutant HSPB1^81^ and cultured DRG neurons expressing mutant MFN2, in which fragmented and abnormally clustered mitochondria and altered axonal transport are reported^82^. Additionally, exogenous supplementation of atypical deoxy-SLs (the levels of which are elevated in HSN1 cells) to primary DRG cells has been shown to result in mitochondrial swelling^83^. HSN1 iPSC-derived sensory neurons have been shown to endogenously produce neurotoxic DSBs^28^; however, the presence of mitochondrial phenotypes has yet to be confirmed. Recently, HSPB1 mutations were shown to alter mitochondrial import, suggesting that the defective mitochondrial phenotypes observed in disease models of CMT2F could stem, at least partially, from disrupted mitochondrial function^84^. The presence of mitochondrial deficits in IPNs and the fact that length-dependent degeneration occurs in several axonopathies in which the longest axons are affected earlier and more severely highlight the importance of investigating cellular processes, especially within the axonal region.

### Investigating the axonal region/length: microfluidic devices and spheroids

Due to the length of peripheral neurons, which can reach 1 m in adults, specific energy, transport, and metabolic requirements are essential for effective functioning. These requirements make peripheral nerves particularly vulnerable to compromised cellular processes and toxic insults.

Therefore, being able to mimic the length of nerves by distinguishing the cellular processes between the soma and axons would be very relevant for studying IPNs. Within the axonal region, a distinction between the proximal and distal segments can be useful, e.g., to systematically investigate the effects of length on axons^85^. Butler et al. demonstrated the relevance of the proximal and distal axon regions in CMT2A human embryonic stem cell (ESC)-*MFN2*^R94Q^-derived motor neurons. Three regions along the axon located 100–300 µm, 900–1100 µm, and 1600–2000 µm from the cell body were distinguished. Interestingly, *MFN2*^R94Q^ motor neurons have fewer mitochondria per micron in the distal regions of axons. A significantly reduced aspect ratio of mitochondria was particularly prominent in the distal part of the axon compared to that of their wild-type counterparts^86^. These proximal and distal axon regions also showed marked differences in axonal transport between *FUS* and *TDP43* iPSC-motor neurons when MitoTracker and LysoTracker dyes were used^87^. Using microfluidic devices, 2D monolayer iPSC-derived motor neurons can be grown on one side of a microfluidic device, which allows for the study of outgrowths on the other side of the fluidic device, in which potential direction-specific axonal transport phenotypes can be assessed. In addition, location-specific mitochondrial phenotypes can be assessed. Axotomy experiments can assess postaxotomy regrowth and determine whether more severe mitochondrial phenotypes occur in the postaxotomy regrowth neurite area. Microfluidics has also been a powerful platform for preparing axons for RNA-seq and generating unique transcriptional profiles to determine the enrichment of transcripts important for mitochondrial and ribosomal functions^88^.

Additionally, the generation of iPSC-derived spheroids—three-dimensional cellular aggregates of a single cell type—demonstrated that modeling the proximal and distal segments of an axon can facilitate the tracking of axonal transport and the quantification of neurite outgrowth. For the CMT2E subtype, NEFL deposits were observed along the axons, and increased levels of NEFL were released into the supernatant^73^. Interestingly, greater levels of released NEFL were observed in the 3D spheroid cultures than in the corresponding 2D cultured motor neurons. The application of spheroid models is not limited to motor neurons but extends to proprioceptive sensory neurons as well. Researchers have utilized spheroids generated from the same human neural stem cell source to compare motor neuron spheres and proprioceptive sensory spheroids in both healthy and sporadic amyotrophic lateral sclerosis (ALS) human neural stem cells^89^. By using spheroids, one can explore the distinct characteristics and disease mechanisms of different neuronal populations relevant to IPN pathogenesis (even in a specific axon region-dependent manner), providing a more comprehensive understanding of the disease. However, while these models offer the advantage of capturing length-dependent features, a major determinant of disease pathology in patients with axonopathies, it is essential to address the limitations of long-term differentiation in these models. Axons may remain isolated from other cells for extended periods, potentially affecting the realistic nature of these cells. To overcome this challenge, incorporating coculture systems that enable interactions with different cell types represents a valuable approach.

### Cocultures: Schwann cell myelination and NMJ formation

Despite the substantial expansion of iPSC-derived motor neuron research in IPNs, progress in iPSC-derived Schwann cell research has been comparatively limited. A key focus has been the generation of iPSC-derived Schwann cell progenitors, as they fail to effectively myelinate iPSC-derived neurons. Indeed, failure to generate in vitro myelination from primary human Schwann cells cultured with primary human DRG neurons is also well documented^90^. Despite this limitation, promising demyelinating studies have used coculture models consisting of rat Schwann cells and human iPSC-derived peripheral sensory nerves^28,91^. These models have provided important insights; for instance, in HSN1 disease myelinating cocultures, the toxic deoxy-sphingobases released by HSN sensory neurons impact closely associated wild-type Schwann cells. This results in a major disruption of nodal complex proteins after 8 weeks, leading to complete myelin breakdown after 6 months^28^. Another coculture model used to study myelination on a chip is nerve conduction velocities, which are used for evaluating electrophysiological and histological metrics, the gold-standard assessment techniques previously possible only for in vivo studies^92^. However, as all-human PNS coculture models are becoming more feasible due to the establishment of iPSC protocols, researchers should avoid the use of mixed rodent neuronal and human iPSC-Schwann cell cultures due to the possibility of residual endogenous rodent Schwann cells that may persist in, for example, rodent DRG cultures, even after the use of compounds targeting mitotically active cells. The use of genetically labeled Schwann cells will circumvent this and, when using an all-human stem cell system, will help identify transplanted iPSC-Schwann cells from spontaneous Schwann cells that may develop from iPSC-sensory neurons. Moreover, the use of genetic labels is essential when incorporating iPSC-Schwann cells into in vivo rodent sciatic nerve injury models. Recently, Mukherjee-Clavin and colleagues validated the presence of MBP-positive myelin segments in Schwann cells from iPSCs cocultured with iPSC-sensory neurons for 2–5 months^50^. In another recent study, researchers successfully differentiated iPSCs into Schwann cells within 21 days using a defined serum-free medium. When cocultured with iPSC-derived motor neurons, these SCs displayed myelin-related marker expression, migrated through a porous collagen sponge, and formed myelin sheaths around motor axons after 8 weeks^93^.

Another important and poorly investigated hallmark that can be studied using coculture models is the degeneration of neuromuscular junctions (NMJs), which bridge peripheral nerves and muscles. Several transgenic CMT mouse models have demonstrated NMJ loss, dysfunction, or morphology differences^94–96^. However, the morphology, active zone protein localization, and molecular composition of human NMJs clearly differ from those of mouse NMJs^97^, further stressing the importance of creating a human NMJ model reflecting human-specific biology. Since the differentiation of skeletal muscle from iPSCs remains challenging, coculture models for human NMJ studies often use a combination of iPSC-derived motor neurons with primary human differentiated myotubes^98,99^. These models can be generated in 2D or 3D microfluidic devices or as motor neuron spheres overlayed on top of differentiated myotubes. Myoblast determination protein 1 (MyoD)-iPSC inducible protocols have also been shown to be successful in generating differentiated myotubes. These muscles, along with motor neurons derived from the same iPSCs, have been used in a neuromuscular model to study myasthenia gravis (MG)^100^. For CMT research, ‘degeneration’ may not always be evident, especially in iPSC-motor neurons grown in isolation without coculture with muscle cells. However, by employing coculture systems that include muscle cells, degeneration and its interplay with motor neurons might be better examined. Recent findings in CMT2D have shed light on the significance of studying the interplay between motor neurons and muscles. BDNF/TrkB impairments were found to correlate with transport disruption and overall CMT2D neuropathology. Supplementation of muscles with BDNF restored normal axonal transport in neuropathic mouse models^101^, indicating that in vitro cocultures could serve as a valuable platform for investigating these interactions.

### iPSC-derived organoids and human peripheral assembloids

While multiple human CNS organoid models have been established^102,103^, even for studying myelination^104^, the use of organoids focused on the PNS has been underexplored. In contrast to the previously discussed spheroids, organoids represent more complex structures consisting of multiple cell types that self-organize. Recently, iPSC-derived sensorimotor organoids containing motor neurons and skeletal muscle, along with sensory neurons, astrocytes, microglia, and vasculature, were characterized^105^. Determining within a single organoid whether motor neuron diseases predominantly spare sensory neurons while sensory disorders, such as HSANs, do not typically affect motor neurons would be facilitated by this model. An adapted protocol also described the presence of myelinating Schwann cells and demonstrated CMT1A myelin defects, such as hypermyelination of the smallest axons and recapitulation of increased myelin periodic distance^106^.

Although organoids are a model that closely mimics in vivo complexity and are composed of many different cell types, this in vitro model provides no directional cell growth. This in vitro situation differs from the in vivo situation, where the PNS stretches in a directional manner from the spinal cord toward the most distal parts of the limbs. Therefore, NMJ-focused studies are challenging to perform because different cells are located throughout the organoid, which does not reflect the length-dependent situation required for axon–NMJ interactions.

To resolve this shortcoming, modeling separate neural and muscle compartments may provide more directional growth of the axon from the motor neuron body toward the muscle, thereby facilitating these experiments. Recently, a 3D iPSC-derived neuromuscular organoid model was developed through a neuromesodermal progenitor stage^107^. These complex models demonstrated the presence of component tissues, maturation, and functional NMJs supported by terminally differentiated Schwann cells. Interestingly, defects in NMJ integrity and reduced contractile activity of the muscle were observed after treatment with autoantibodies from MG patients. An adaptation of this approach could be the generation of neuromuscular assembloids, in which motor neuron spheres are fused with differentiated human muscle spheres. However, the timing of sphere/organoid assembly may vary between different models. In contrast to iPSC-derived neuromuscular organoids, a neuromesodermal assembloid model was recently developed. iPSC-differentiated Sox1^+^ neuroepithelial cell spheres were fused to mesodermal cell spheres, creating a neuromesodermal cellular interface^108^. With this model, intercellular crosstalk between both tissue compartments, including neural crest cell induction and migration, was observed. Axons projecting from the mesodermal compartment were associated with Schwann cells, and nerve fibers were observed to interact with the codeveloping vascular plexus. Assembloids are advanced models in which different cellular components are combined to create a more intricate representation of tissue organization and interaction.

Complex iPSC models are now available for HSAN patients who clinically present with an inability to sense pain and temperature, leading to different injuries, such as ulcerations^109^. Currently, most human innervated skin models are limited in composition and are often composed of ex vivo human donor skin explants. However, in a recent study, a human 3D in vitro innervated skin model was developed using a scaffold to obtain a better biomimetic composition. The skin component consisted of primary human keratinocytes and human fibroblasts, while the neuronal component was composed of iPSC-derived sensory neurons. The study demonstrated that the dermal component consisted of fibroblasts and synthesized collagen and that the sensory neurons reached the epidermis, which suggested this would be a useful model for studying neuron-skin interactions^110^. In another study, an innervated human skin model was developed; however, iPSC-derived Schwann cells were added to the culture for iPSC-derived sensory neurons to reach the epidermis^111^. This model could be useful for studying cutaneous neuroinflammation.

**Fig. 4:**
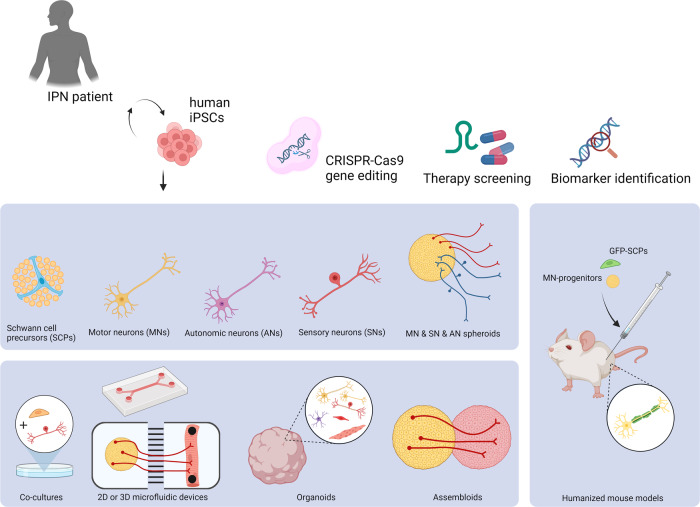
Current stem cell models used to study inherited peripheral neuropathies (IPNs). The figure shows various stem cell models. The upper blue panel represents monocultures, while the lower panel illustrates more complex models, from 2D to 3D cocultures, grown in microfluidic devices or as neuromuscular assembloids or organoids containing multiple cell types. The right blue panel depicts the potential use of these models in combination with mice (known as humanized mouse models). Finally, the figure also highlights the ability of these models to contribute to the assessment of therapeutics and gene-editing approaches or the identification of potential biomarkers. Created with BioRender.com.

The 2D and 3D coculture, assembloid, or organoid approaches we have described for IPN models could be used in complementary experimental designs, as each model presents distinct advantages (Fig. 4)^102^. 2D cultures offer benefits such as homogeneity, uniform nutrient access, and scalability, making them better for (high-throughput) genetic and pharmacologic screens. However, 3D cultures better represent essential cellular interactions and replicate structural features. The coculturing or assembly of two or more multiple cell-type stages can involve different genetic backgrounds, treatments, or gene-editing experiments. For example, it is now possible to address the effect of a gene mutation in one population (e.g., motor neurons) on all other included cell types. The opposite would also be possible, allowing the study of the primary cause, e.g., neuromyopathies. From a technical standpoint, the editing or transduction of cell spheres before assembly offers the ability to answer questions after the impact of coculturing. For instance, motor neuron-specific transduction with optogenetic constructs enables the stimulation of motor neurons to assess NMJ functionality through muscle contraction. However, in iPSC-derived neuromuscular organoids, motor neurons, skeletal muscle cells, and Schwann cells codevelop and interact in the system from the earliest stages of development. These models might be better suited for studying the contribution of each cell type at different stages of development and maturation. Human-derived 2D and 3D (co)cultures, organoids, and assembloids can also be used in the validation of biomarkers of disease progression to support their translational potential. Recently, reduced *PFN2* expression was observed in motor neurons differentiated from CMT2 patient-derived iPSCs and sciatic nerves of CMT2 mice compared with controls^112^. Similarly, in the case of CMT2E, the presence of neurofilaments in the culture media supernatant of patient motor neurons has been identified as a potential in vitro biomarker for axonal degeneration^73^. More complex in vitro models incorporating other cell types, such as muscle cells, may be used to validate recently identified CMT biomarkers, such as GDF15 or NCAM1^113^. Elevated NCAM1 has been observed in various mouse models and patients and may serve as an indicator of disease severity by correlating positively with the CMTES neuropathy severity score. The increase in NCAM1 may reflect muscle regeneration triggered by denervation, further emphasizing the need to explore these dynamic processes in more complex in vitro models.

## The next generation of in vitro models for IPNs: immune cell incorporation and complex interactions

Despite the expanding developments in human cellular models of neurodegenerative diseases, these models are limited in terms of maturation and complexity. The lack of complexity includes complex neuronal circuits, cell–cell interactions, glial complexity, and the absence of vascular and immunological components. These limiting components known to act in neuromuscular diseases are essential for improving the assessment of the effectiveness of a potential clinical trial compound or therapy tested in vitro. Additionally, the lack of complexity could also explain why very mild or no neurite degeneration has been reported for CMT phenotypes across different studies^17,21^.

Coculture or organoid models have already been demonstrated to be more physiologically relevant systems, enabling translational applications. However, the inclusion of immune cells in in vitro models remains rare, despite several studies demonstrating immune cell activation and infiltration in degenerating peripheral nerves^114,115^. In the context of CMT1, there is clear evidence of immune cell involvement in disease pathogenesis. For example, in 6-month-old sciatic nerves of a CMT1B MpzD61N/+ mouse model, macrophages were observed to engulf myelin debris, and an increase in mast cell numbers was noted. PNS macrophages can also adopt pathogenic functions, especially when activated in nonlesioned nerves^116,117^. The cytokine CSF-1 has been identified as a key molecule in the activation of nerve macrophages. Targeting the CSF-1 receptor with an inhibitor has shown promising results in substantially improving the disease course in CMT1B and CMT1X mouse models. Recent studies have also shown that Schwann cells contribute to myelin debris clearance through autophagy (myelinophagy) after nerve injury and aberrant autophagy induction has been observed in a mouse model of CMT1A^118^ and inflammatory segmental demyelinating neuropathy^119,120^. A recent study revealed robust upregulation of markers of Schwann cell autophagy after injury and in genetically mediated neuropathy when nerve macrophages were pharmacologically depleted^121^. These findings reveal novel communication and interactions between Schwann cells and macrophages and again highlight the complexity of the interplay between different cell types in the disease process.

In pediatric CMT1 patients, electron microscopy studies have revealed the increased presence of endoneurial macrophages, which are associated with active demyelination^122^ and strongly suggest the involvement of immune cells in the pathogenesis of CMT1^123^. Furthermore, the response of a subgroup of patients to anti-inflammatory treatment and the presence of inflammatory infiltrates in their peripheral nerves provide additional evidence supporting the involvement of immune cells in the disease^122,124,125^. In the case of axonal CMT, mutations in *GDAP1* have been associated with increased reactive oxygen species (ROS) production and the presence of inflammatory mediators, such as TNF-α and proteins of the complement system^126^. These findings indicate a complex interplay between immune responses and axonal dysfunction in this subtype of CMT. Additionally, iPSC-derived Gdap1-null motor neurons were found to induce a fragile phenotype in MNs, characterized by mitochondrial dysfunction and activation of the innate immune response^127^. Another recent study demonstrated that in both demyelinating and axonal CMT patient sera, a significant increase in serum complement system protein levels, including C1q and C3, was observed compared to levels in control sera^113^. Increased C1q and C3 deposition was also detected on the sarcolemma, in part colocalized with the NMJs, of two genetically distinct CMT patients (FIG4 and GDAP1), suggesting that the upregulation of complement proteins could at least partially arise from NMJ degeneration.

Moreover, in the context of IPNs, there is growing interest in exploring other potential drivers of inflammation. One intriguing area of research involves investigating mitochondrial DNA (mtDNA) release and cGAS/STING activation, which has recently been implicated in neurodegenerative diseases such as ALS^128^ and is currently gaining attention^129^. Studies have shown that mtDNA is released through macropores formed on the outer membrane of mitochondria via VDAC oligomerization, particularly in response to mitochondrial stress. Although the involvement of mtDNA release and cGAS/STING activation in IPNs requires further investigation, it is worth noting that mitochondrial dysfunction is a reported hallmark shared among various IPN disease-causing genes^21,83,84^. This finding suggests a potential link between mitochondrial stress and inflammation and IPN pathogenesis.

Considering the potential advantages of incorporating immune cells into iPSC-derived neuronal cultures, it is also crucial to acknowledge the existing limitations and challenges associated with this approach. One major limitation arises from the inherent heterogeneity of immune cell populations, which encompass a diverse array of cell types, each with distinct functions and responses. Integrating this diversity into in vitro models requires careful consideration of the specific immune cell types relevant to the neuropathy under investigation. Maintaining the appropriate ratios and interactions among neurons, glial cells, muscles, and immune cells adds an additional layer of complexity.

Despite these challenges, the immunologic component of CMT1A has recently been investigated in vitro using three congruent patient-specific cell types (human iPSCs, human ESCs, and direct lineage-converted cells). Commonly dysregulated inflammatory signaling pathways were described, along with two proteins, CXCL1 and MCP-1, which were found to be commonly upregulated among all three congruent models. Both CXCL1 and MCP-1 are potent chemokines that orchestrate the recruitment and activation of specific immune cells to sites of inflammation or injury. To study whether this increase in cytokine expression is sufficient to induce monocyte chemoattraction, a transwell migration assay was performed, which revealed greater human THP-1 monocyte migration toward CMT1A SCP-conditioned medium than to control-conditioned medium^50^. Furthermore, sural nerve biopsy samples were obtained from two CMT1A patients, and sample analysis showed significantly increased levels of the CXCL1 and MCP-1 proteins compared with those in samples obtained from controls.

This finding suggests that to better recapitulate CMT phenotypes, more complex in vitro models may benefit from the inclusion of immune cells and potentially other cell types to provide a better understanding of non-cell-autonomous phenotypes in IPNs. The microfluidics field presents exciting possibilities for developing advanced models that integrate multiple cell types on a single chip, including peripheral neurons, myelinating Schwann cells, immune cells, and muscle cells, allowing the study of intricate interactions. This phenomenon was recently explored for ALS, with iPSC-derived astrocytes from *FUS*-ALS patients incorporated into a human motor unit microfluidics model to investigate the effect of astrocytes on iPSC-derived motor neurons and functional NMJs^99^. This study demonstrated that the dysregulation of astrocyte homeostasis resulted in a FUS-ALS-mediated increase in the reactivity and secretion of inflammatory cytokines. Coculture of these iPSC-derived astrocytes from FUS-ALS patients with motor neurons and myotubes led to a cytotoxic effect on motor neuron–neurite outgrowth, NMJ formation, and functionality, all of which were improved or fully rescued by isogenic control astrocytes, underscoring the role of more complex models.

Moreover, the development of a ‘blood-nerve-barrier’ (BNB) on a chip, building off recent developments for the ‘blood‒brain-barrier’ (BBB)^130^, would significantly contribute to personalized medicine in patients with IPNs. Such neurovascular chips could accurately predict the permeability of peripheral nerves to pharmacological agents or therapeutic approaches.

In conclusion, these advanced models may offer a promising strategy for studying the complex interplay that occurs among nerve cells, glial cells, muscles, and immune cells, ultimately leading to increased understanding and targeted therapies for IPN patients.

## Future perspectives for IPN-focused stem cell research: challenges and strategies

### Overcoming variability in iPSC research for IPNs

One drawback of iPSC research is that different iPSC lines can show considerable phenotypic variation, which could hamper the integration of data across research groups or impact the replication of key findings. Variation could arise from several factors. The reprogramming process itself can be influenced by variations in starting cell populations, such as different types of somatic cells or cells from different individuals^131^, leading to differences in reprogramming efficiency and resulting in differences in iPSC quality. However, this is also true for the choice of reprogramming factors or delivery methods. Once iPSCs are generated, their differentiation into specific cell types introduces another layer of variability, with different protocols leading to different purities or different specific cell-type identities. Intercellular variability within iPSC lines can also exist due to factors such as clonal heterogeneity and epigenetic variations. To overcome some of these challenges, research standard initiatives are being established (https://www.isscr.org/standards). Advances in genome editing techniques can now enable the generation of isogenic controls through the correction of pathogenic mutations in patient-derived iPSCs or the introduction of the desired mutation into a control iPSC line, preserving the genetic origin.

A recent initiative addressed this by characterizing a reference ‘KOLF2.1J’ iPSC line. The line showed genomic stability after highly efficient CRISPR gene editing, the integrity of the p53 pathway, and the efficiency of differentiation toward multiple lineages, as confirmed by multiple research groups^132^. Currently, this iPSC line is used in an initiative from the NIH Center for Alzheimer’s and Related Dementias to derive hundreds of gene-edited and functionalized subclones to be distributed widely throughout the research community, along with associated datasets, with the aim of promoting the standardization required for large-scale collaborative science in the stem cell field. As the IPN field involves more than 1000 mutations in more than 100 distinct disease-associated genes, the field could also benefit from such initiatives. For many laboratories, the threshold to start working with iPSCs is still significant due to the cost or lack of experience in generating and differentiating these lines. Extending this approach to the IPN field will provide more researchers with access to iPSCs with appropriate controls, thereby addressing these barriers and enabling broader usage (and standardization) of iPSCs in IPN research.

### Immaturity of iPSC-derived models in the context of IPNs

The immaturity of human iPSC derivatives poses a challenge in the context of IPNs, as they often resemble cells at a more fetal than adult stage. The utility of the platform is therefore likely to depend on the exact genes being studied and the maturity of the cells that have been modeled. A good example is voltage-gated sodium channels (Nav) in sensory neurons generated from the Chambers protocol. Nav1.7 is well expressed, and the fact that biallelic loss-of-function Nav1.7 mutations associated with congenital insensitivity to pain result in hypo-excitability emphasizes the role of this channel in the regulation of sensory neuron excitability. In contrast, Nav1.8, which is a key marker of native adult nociceptors, shows very little functional expression in these neurons (which express Nav1.5, a marker of fetal sensory neurons)^38^; these neurons would therefore be a poor model for screening therapeutics directed at this channel or its mutants. Incomplete development may limit the ability of these neurons to accurately recapitulate mature IPN disease phenotypes.

To address this limitation, ongoing efforts are focused on increasing maturation by using various strategies, including extended culture periods, stimulation approaches, tissue engineering approaches, and attempts to better mimic the in vivo microenvironment. Another strategy is to bypass the intermediate iPSC stage through the direct conversion of fibroblasts into neurons or glia. This approach is suitable when epigenetic markers such as those associated with aging are retained. As such, this approach could be considered a tool for modeling late-onset diseases. This suitability has also been shown in CMT1A, where CMT1A patient fibroblasts were directly reprogrammed into a Schwann cell lineage via a neural crest intermediate^50^. These directly reprogrammed Schwann cells indeed showed enrichment of aging-related genes and features of DNA damage compared to iPSC-differentiated Schwann cells. Kim et al. demonstrated that human skin fibroblasts can be directly induced to differentiate into integration-free SCPs using episomal vectors (Oct3/4, Klf4, Sox2, L-Myc, Lin28, and p53 shRNA), and these cells passed the same characterization and functional assessment tests as those from the previously reported iPSC-SCP model^48^. Another study converted human fibroblasts of patients carrying dominant and recessive GARS mutations to induced neuronal progenitor cells. These neuronal cells, in contrast to fibroblasts itself, exhibited reduced mitochondrial respiratory chain enzymes and significant defects in mitochondrial proteins^133^. Additionally, for CMT2A, patient fibroblasts are directly reprogrammed into motor neurons via microRNA-mediated neuronal conversion, resulting in mitochondrial fragmentation^134^. However, this approach is associated with restricted cell fate options, limited efficiency, and incomplete conversion, which might result in a heterogeneous population of cells with varying degrees of neuronal characteristics. Direct reprogramming of fibroblasts has also been suggested as a means to generate nociceptors; this process was previously performed on fibroblasts from patients with familial dysautonomia (HSAN-III)^135^ and led to reduced outgrowth and branching in neurons derived from individuals with familial dysautonomia. However, the efficiency of this process is low, so it has not been widely adopted.

Finally, alternative-source stem cells, such as dental pulp stem cells (DPSCs), are promising for studying IPNs. Dental pulp stem cells (DPSCs), which are derived from tooth pulp, have a neural crest origin and are developmentally linked to the Schwann cell lineage, which highlights their propensity to differentiate into Schwann cells with a relatively mature phenotype and makes them good candidates for studying demyelinating IPNs^136,137^. DPSCs express glial markers, secrete neurotrophic factors, and can myelinate neurites in 3D constructs. DPSCs can also become functional neurons and are easily obtained from wisdom tooth extractions. Notably, adult stem cell sources have limitations such as variable quality. Nevertheless, combining different methods can advance our understanding of inherited peripheral neuropathies.

### Use of stem cells as preclinical models for testing therapeutic approaches

Since new medicines do not need to be tested in animals to receive U.S. Food and Drug Administration (FDA) approval^138^, stem cell research will become a more prominent platform to test, develop, or validate therapeutic approaches and could contribute to the acceleration of translating drugs or gene therapies into clinical trials. Recently, this iPSC technology has led to a few clinical trials of drug candidates for ALS^139–142^.

The use of iPSC-derived neuronal models is also becoming a promising platform in IPN research for testing therapeutic approaches. For CMT2A specifically, mitofusin agonists could be relevant^143^. Recently, a follow-up study demonstrated that pharmacological activation of endogenous normal mitofusins using a small molecule called MiM111 improved the structure and fitness and increased the movement of mitochondria in both CMT2A mouse nerve cells and in directly reprogrammed CMT2A patient fibroblast motor neurons^134^. As mitochondrial dysfunction has been reported in several iPSC-neuron IPN studies, a mitotherapeutic screen recently performed on primary neurons might also be a good strategy for application in iPSC-IPN research^144^. This screen could be combined with a high-content screen for axonal transport assessment^145^ and facilitate deeper insights into potential therapeutic targets.

Some of the most studied compounds tested on iPSC-derived motor neurons are HDAC6 inhibitors, which are known to alleviate mitochondrial transport defects in models of neurodegenerative diseases^20^. First, HDAC6 inhibitors restored tubulin acetylation and consequently increased axonal transport and the total number of mitochondria in three CMT2 mouse models. Later, HDAC6 inhibitors were shown to improve phenotypes in iPSC-derived motor neurons of CMT2D and CMT2F patients^67,146^ as well as in human ESC-CMT2A-derived motor neurons^86^. Due to CMT heterogeneity, a recent study investigated the possibility of targeting common pathomechanisms in different CMT2 subtypes. This finding showed that a dual leucine kinase (DLK) inhibitor was able to ameliorate mitochondrial transport, morphology, and functionality deficits in both *MFN2*^R94Q^ and *NEFL*^P8R^ patient motor neuron lines^21^. The use of other kinase inhibitors has also been shown to improve disease phenotypes; for example, fewer NEFL deposits were observed in kinase inhibitor-treated *NEFL*^N98S^ patient motor neurons than in their untreated counterparts^73^.

More recent work by Majd et al. showed that iPSC-derived Schwann cells can be utilized for drug discovery applications^47^. Here, two different methods for generating Schwann cells showed that these iPSC-derived Schwann cells could functionally myelinate in vitro and in vivo when transplanted into a rodent sciatic nerve injury model^47^. Using these iPSC-derived Schwann cells, the authors identified Schwann cells as being selectively vulnerable to glucotoxicity and performed a high-throughput screen on these cells to identify disease-modifying candidate compounds for diabetic neuropathy patients^47^. More complex models have also shown their relevance in assessing therapeutics. Cocultures of myelinating rat Schwann cells with iPSC-derived sensory neurons confirmed that L-serine supplementation not only reduced deoxysphingolipid base (DSB) synthesis but also increased axon outgrowth and the expression of nodal proteins^28^. Recently, iPSC-derived organoids containing myelinating Schwann cells were generated and characterized for CMT1A neuropathy^106^, the subtype accounting for the majority of IPN cases. Combinatorial treatment with baclofen, naltrexone, and sorbitol (currently in phase III clinical trials such as PXT3003, Pharnext, Inc.) was shown to be beneficial in CMT1A rats^147,148^ and in iPSC-derived organoids, providing the first evidence in a human system that these compounds ameliorate the pathological features of CMT1A neuropathy^106^.

Human iPSC models are particularly powerful in the context of genetic therapies where the therapeutic strategy is specific to the human genome. Gene therapy strategies depend on the specific mode of inheritance^149^. Recessive disorders can be effectively treated by gene replacement strategies that can be effectively tested in animal models, as shown by the development of Spinraza for SMA^150^. In the context of IPNs, most IPNs are dominantly inherited, with a minor proportion of IPNs being autosomal recessive or X-linked recessive. Dominant diseases often require an alternative genetic strategy, such as knocking down the expression of a mutant gene product. Notably, for IPN treatments, the approval of patisiran (RNAi) and inotersen (ASO) for the treatment of hereditary transthyretin amyloidosis (ATTR) marked a significant milestone in this regard^4,5^. Robust human model systems are ideal for testing this category of therapeutics designed to interact with the human genome. Although humanized animal models can be developed to test individual target genes, the remaining non-human genetic background makes the evaluation of genetic off-targets problematic.

In the case of CMT2A, a study employed iPSC-derived motor neurons, in which a single vector combines an RNAi component to silence a harmful gene and a cDNA component encoding a normal, RNAi-resistant version of the corresponding mutated protein^151^. This approach successfully restored the characteristic MFN2 mutant mitochondrial abnormalities. Furthermore, gene therapy has also been performed in more complex iPSC models, such as CMT1A iPSC-derived organoids, in which a shRNA directed toward reducing *PMP22* gene expression rescued myelin deficits^106^.

Another promising approach involves targeted inactivation of dominant disease alleles through gene editing. In contrast to antisense and RNA interference approaches, which require prolonged activity of the vector, gene editing requires only transient expression of the therapeutic molecule to result in a permanent effect. Using CRISPR/Cas9, a recent study successfully inactivated the CMT2E disease *NEFL* allele in patient-derived iPSCs. This introduction of a frameshift at the pathogenic N98S mutation led to effective knockout of mutant gene expression and amelioration of the disease phenotype, comparable to that seen in an isogenic control with precise mutation correction^25^. A number of dominant IPNs could be amenable to this approach. Such strategies could be particularly interesting for diseases caused by mutations in small heat shock proteins (HSPBs), as molecular compensation is very likely to occur within the small heat shock protein family. Knock-in and knockout CMT2L mouse models have provided intriguing insights^152^. For example, *Hspb8*^K141N/K141N^ knock-in model mice mirror the clinical features of patients, while Hspb8^−/−^ mice show no motor deficits or electrophysiological abnormalities^152^. Hspb1 knockout mice do not present with any overt phenotype either^153,154^, further suggesting (partial) redundancy among HSPB functions. When considering gene-editing approaches for HSPBs, whole-gene knockout of a small heat shock protein might present a more straightforward option than an allele-specific approach. An antisense oligonucleotide (ASO) targeting wild-type HSPB1 mRNA (OGX-427) is already in clinical trials for cancer treatment^155^, making it a candidate for testing in CMT2F HSPB1-related diseases.

Challenges in gene therapy for IPNs include precision targeting, ensuring long-term efficacy, establishing effective delivery methods, addressing immune responses, and minimizing off-target effects. The vast number of mutations associated with IPNs creates a challenge for gene therapy since targeting each dominant mutation individually is not feasible. Notably, the vast majority of gene-editing studies are performed in dividing cells that can be transfected in vitro or ex vivo. The DNA repair mechanisms involved in gene editing are likely to differ between Schwann cells and postmitotic neurons. This area of biology is understudied, and iPSC-derived models provide an opportunity to greatly expand our knowledge. Delivery to differentiated cells in vivo also remains a challenge because of considerations such as efficiency, cell-type selectivity, immunogenicity, and the duration of activity of the payload^156,157^. Complex iPSC models incorporating different cell types, including peripheral nerves and myelinating Schwann cells, hold great promise for testing these approaches in a human genetic setting, which is otherwise very challenging. Furthermore, these complex models might also help in the assessment of precise targeting and immune responses in the context of gene therapies. In summary, iPSC models show great promise for various aspects of therapeutic development, but animal models remain crucial for evaluating in vivo delivery, bioavailability, biodistribution, and other pharmacological properties that are challenging to predict in vitro.

## Concluding remarks

In conclusion, the potential of human stem cell models in IPN research is promising. Over the last decade, various disease mechanisms in different IPN patient subtypes have been successfully recapitulated in iPSC-derived neuronal models. Moreover, the development of more complex in vitro cultures, such as cocultures, human-derived organoids, and assembloids, has brought us closer to mimicking physiological complexity and shows remarkable similarities to in vivo systems and tissues. The use of these different neuromuscular systems (either 2D or 3D) will simplify the bulk testing of compounds and gene therapies, which is less straightforward in the few transgenic mouse models that are currently available for IPNs. However, considerable effort is still needed to further improve our models and phenotyping methods. By continuously refining these techniques, we can assess potential therapeutics in 2D monolayer-derived neurons first, followed by more sophisticated in vitro cultures, ultimately contributing to the establishment of effective treatments for IPNs.
